# Predictors of prognosis by cardiac magnetic resonance imaging in patients with idiopathic restrictive heart disease

**DOI:** 10.1186/s43044-025-00657-9

**Published:** 2025-06-16

**Authors:** Ahmed Shawky Asfour, Alaa Mohamed Abd Al-Kader Mohamed, Hesham Yahia Abd Al-Salam, Heba Mostafa Ahmed Aboelsoud, Mahmoud Shaaban, Mohamed Zaki Elramly

**Affiliations:** 1 Cairo University, Giza, Egypt; 2 Tanta University, Tanta, Egypt

**Keywords:** Restrictive cardiomyopathy, Cardiac MRI, Global longitudinal strain, Late gadolinium enhancement

## Abstract

**Background:**

Restrictive cardiomyopathy (RCM) is characterized by increased myocardial stiffness, impaired diastolic filling, and preserved systolic function until advanced stages. Cardiac magnetic resonance imaging (CMR) provides precise strain analysis and tissue characterization, yet its prognostic value in idiopathic RCM remains underexplored. This study aims to evaluate the prognostic significance of CMR parameters, particularly myocardial strain and late gadolinium enhancement (LGE), in predicting outcomes in patients with idiopathic RCM.

**Results:**

Patients demonstrated moderately impaired strain values, with global longitudinal strain (GLS) measured at −10.59 ± 4.64% and global circumferential strain (GCS) at −14.50 ± 4.98%, while maintaining preserved biventricular ejection fractions. LGE was present in 40% of patients. A GLS value greater than −9.5% independently predicted mortality (odds ratio [OR]: 1.195, *p* = 0.044) and heart failure (HF) hospitalization (OR: 1.152, *p* = 0.013). Additionally, LGE emerged as a strong independent predictor of both mortality (OR: 6.340, *p* = 0.004) and HF hospitalization (OR: 4.654, *p* = 0.001).

**Conclusions:**

CMR is a valuable tool for prognostication in idiopathic RCM. GLS and LGE are independent predictors of adverse outcomes.

## Background

Restrictive cardiomyopathy (RCM) is a myocardial disorder characterized by increased myocardial stiffness, impaired diastolic filling, and preserved systolic function until advanced disease stages. Unlike other cardiomyopathies, RCM is primarily defined by diastolic dysfunction, leading to elevated filling pressures, bi-atrial enlargement, and progressive heart failure (HF). The condition can be idiopathic or secondary to infiltrative (e.g., amyloidosis, sarcoidosis), storage (e.g., Anderson-Fabry disease), or systemic (e.g., scleroderma) disorders. Early identification and risk stratification are essential, as RCM carries a poor prognosis with limited treatment options beyond supportive care and heart transplantation [1].

Cardiac imaging plays a crucial role in the diagnosis and risk stratification of RCM. Transthoracic echocardiography remains the first line imaging modality, providing insights into bi-atrial enlargement, restrictive filling patterns, and normal or mildly increased wall thickness. However, echocardiographic parameters alone often fail to provide comprehensive myocardial tissue characterization or quantify the extent of fibrosis. Given these limitations, cardiac magnetic resonance (CMR) has emerged as the gold standard for assessing myocardial structure, function, and tissue properties [2].

CMR enables precise quantification of myocardial deformation parameters such as global longitudinal strain (GLS), global circumferential strain (GCS), and global radial strain (GRS), offering a more sensitive evaluation of subclinical myocardial dysfunction. Additionally, late gadolinium enhancement (LGE) serves as a key biomarker for myocardial fibrosis, correlating with disease severity and prognosis. Prior studies have demonstrated the prognostic utility of GLS in heart failure with preserved ejection fraction (HFpEF), a condition that shares similar pathophysiologic features with RCM. However, data on the prognostic role of CMR-derived strain analysis and LGE in idiopathic RCM remains limited [3, 4].

Therefore, this study aims to evaluate the prognostic value of cardiac MRI in patients with idiopathic restrictive heart disease.

## Patients and methods

### Study design

This is a retrospective cohort study with follow up conducted at the Cardiac MRI unit of Cairo University Hospitals, Egypt. Patients were followed up clinically through regular outpatient visits, telephone calls, and review of medical records over a mean duration of 3 ± 1 years to assess prognostic factors using cardiac MRI in individuals with restrictive heart disease.

### Study population

This study encompassed a cohort of 100 patients aged over 18 years with a confirmed diagnosis of restrictive heart disease and preserved ejection fraction. Patients were excluded if they had ischemic heart disease, cardiomyopathy due to chemotherapeutic agents, constrictive pericarditis, an eGFR < 30 mL/min/1.73m^2^, or pacemakers/implantable cardiac defibrillators (to avoid MRI artifacts).

### Ethical considerations

The study protocol was reviewed and approved by the Ethics Committee of Cairo University Hospitals, Egypt prior to study initiation. Before enrolment in the study, participants were thoroughly informed about the study's objectives, methodology, and a comprehensive risk–benefit assessment. Informed consent was obtained from all individuals. To ensure confidentiality, all collected data remained anonymous, and no identifying information was included in any reports or publications related to the study.

### Clinical evaluation

Comprehensive history, physical examination, and routine laboratory tests were performed, including assessment of comorbidities and systemic signs.

## Procedures

### Echocardiography

Echocardiographic assessments were conducted with all patients positioned in the left lateral decubitus position to enhance image quality. M-mode echocardiography was performed by placing the ultrasound probe in the left parasternal region to evaluate left ventricular (LV) dimensions and wall motion. Two-dimensional (2D) echocardiography utilized standard views (e.g., parasternal short axis, parasternal long axis, and apical four-chamber) to assess LV morphology, function, and regional wall motion abnormalities. Doppler ultrasound was used to measure mitral inflow velocities, the early-to-late diastolic flow velocity (E/A) ratio, E deceleration time, and mitral annular tissue Doppler imaging (TDI) Ea velocity. A restrictive diastolic pattern was identified by an E/A ratio greater than 2, E deceleration time less than 150 ms, and TDI Ea velocity less than 10 cm/s.

### CMR acquisition

CMR was performed using a 1.5 T MRI system with phased-array receiver coils to enhance signal quality, following a routine scan protocol. Using Balanced steady-state free precession (bSSFP) cine imaging, a comprehensive left ventricular assessment was performed through a series of short-axis slices at 1 cm intervals, complemented by three key long-axis orientations: apical four-chamber, three-chamber, and two-chamber. These images were captured with a high temporal resolution of under 45 ms to enhance cardiac motion analysis.

### LGE imaging

LGE imaging was conducted after administering gadolinium contrast (0.15 mmol/kg) 10–15 min before scanning. A 2D segmented gradient echo inversion-recovery sequence captured the same views as cine CMR, with inversion delay times between 280 and 360 ms. LGE extent was visually scored using a 17-segment model, with segments graded on a 5-point scale (0 = no LGE, 4 = 76–100%). LGE extent was assessed as a proportion of the LV myocardium by aggregating regional scores, assigning weights according to the midpoint of each range, and normalizing the total by dividing it by 17.

### CMR-FT

Most patients were in NYHA class I or II at the time of imaging. All were clinically stable and able to undergo MRI without signs of decompensated heart failure, orthopnea, or overt congestion. Strain parameters of the myocardium were extracted from bSSFP cine sequences, which were archived in DICOM format. Image processing was performed utilizing a specialized CMR-FT platform (Segment v4.0, Medviso, segment.heiberg.se) to ensure accurate quantification of myocardial mechanics [5].

At the end-diastolic phase, manual delineation of the epicardial and endocardial contours was carried out to facilitate precise morphological assessment. Subsequently, CMR-FT software autonomously tracked myocardial features throughout the cardiac cycle, facilitating the assessment of deformation across all 16 myocardial segments.

**Fig. 1:**
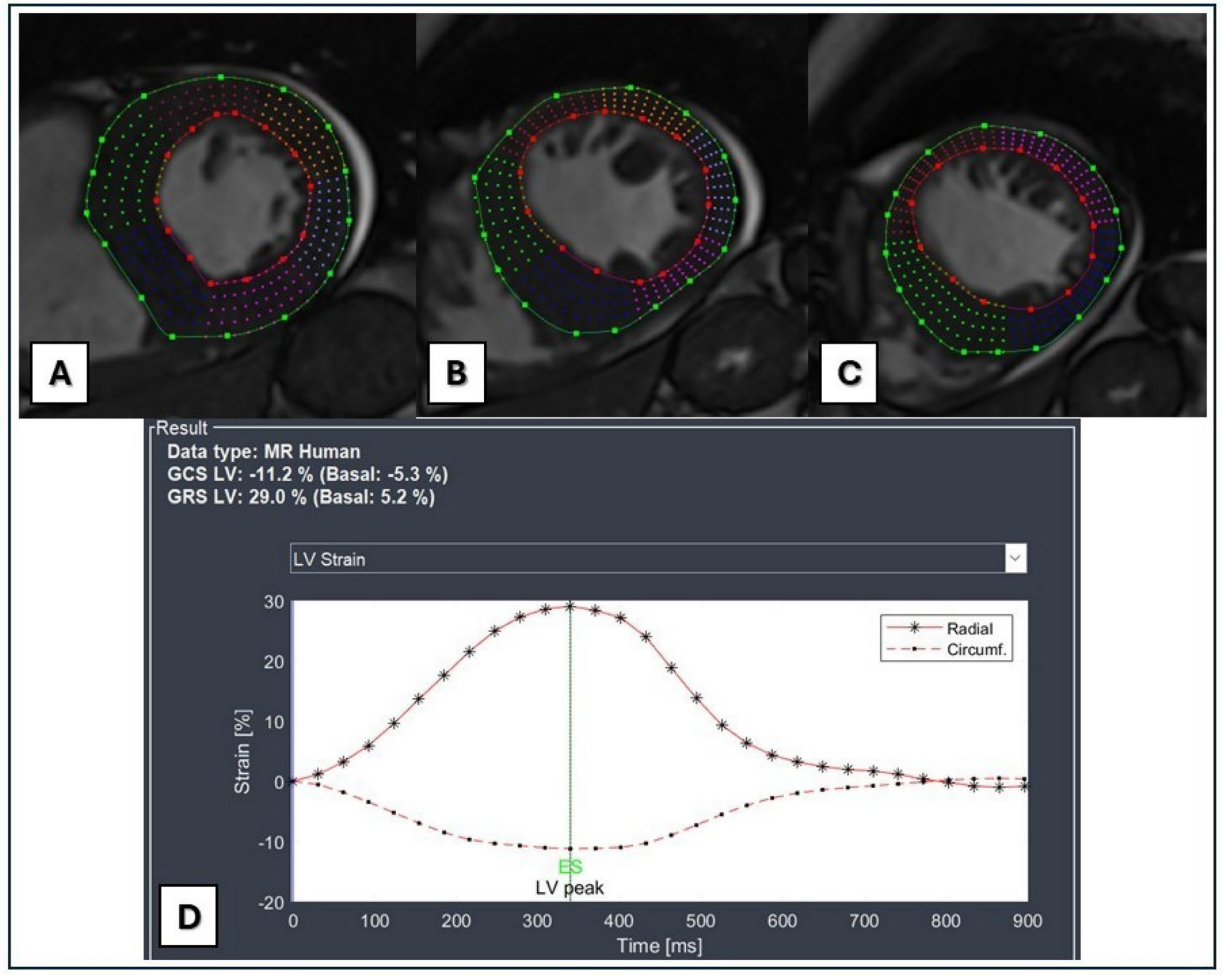
Myocardial strain analysis derived from bSSFP cine short-axis images at the basal (**A**), mid (**B**), and apical (**C**) LV levels. Endocardial and epicardial contours were drawn manually at the end diastolic phase and then the software automatically tracked myocardial deformations in each LV segment throughout the cardiac cycle. **D** Strain–time curves and GCS and GRS values are displayed

**Fig. 2:**
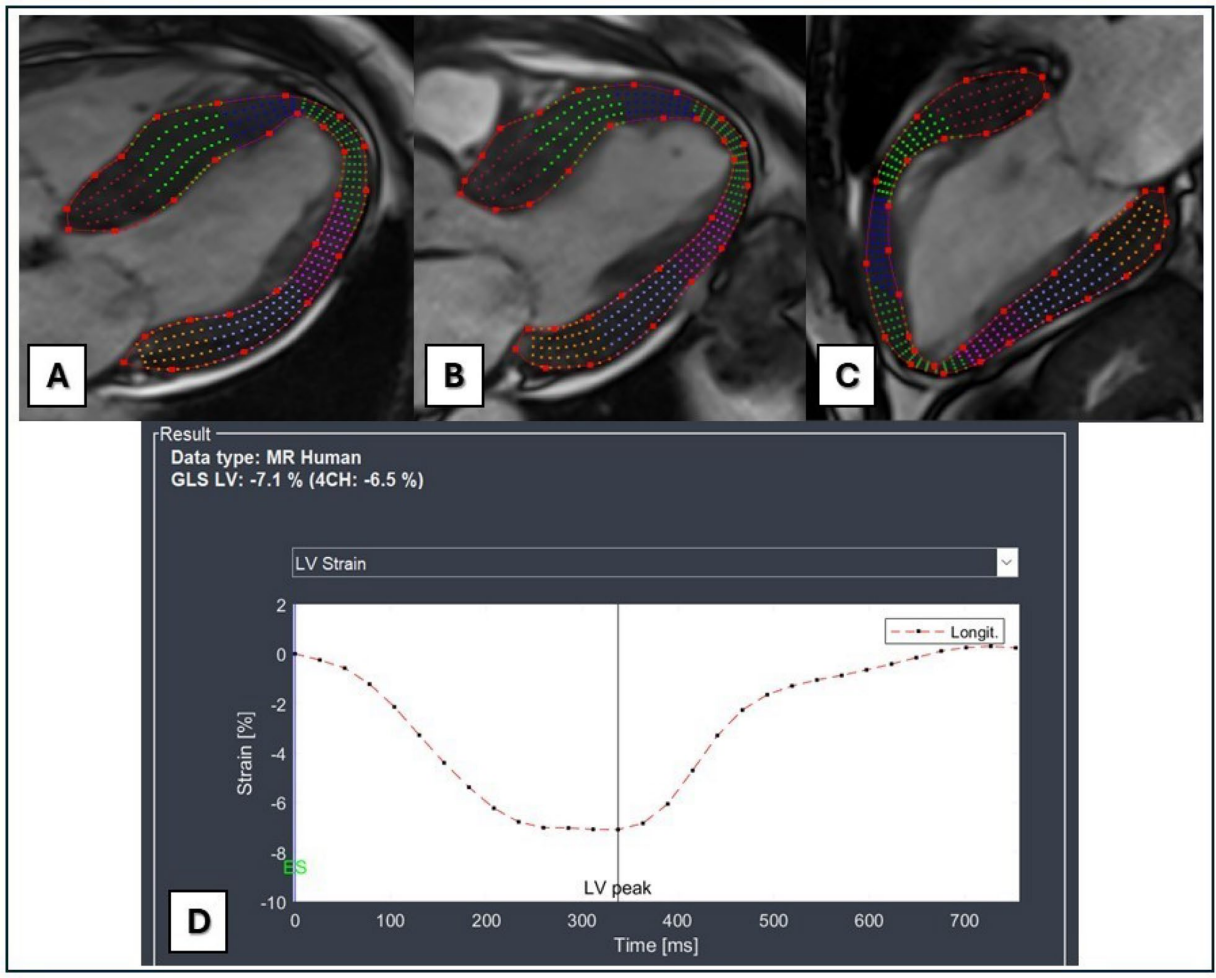
Myocardial strain analysis derived from bSSFP cine 4-chamber (**A**), 3-chamber (**B**), and 2-chamber (**C**) long-axes images. Endocardial and epicardial contours were drawn manually at the end diastolic phase and then the software automatically tracked myocardial deformations in each LV segment throughout the cardiac cycle. **D** Strain–time curves and GLS values are displayed

The LV GLS was calculated from the 4-chamber, 2-chamber, and 3-chamber long-axes images while GCS and GLS values were calculated from the short-axes images Figs. 1 and 2

## Outcomes

The main outcome measure was a composite of hospitalization due to heart failure and all-cause mortality.

### Statistical methods

SPSS version 26 (IBM Inc., Chicago, IL, USA) was utilized for statistical analysis. Quantitative data were reported as mean ± SD and compared between the two groups using an unpaired Student’s t-test. Qualitative variables were expressed as frequencies and percentages (%) and analyzed through Fisher’s exact test, or Chi-square test, depending on applicability. Univariate and multivariate regressions were done for the prediction of mortality and HF hospitalization.

## Results

**Fig. 3:**
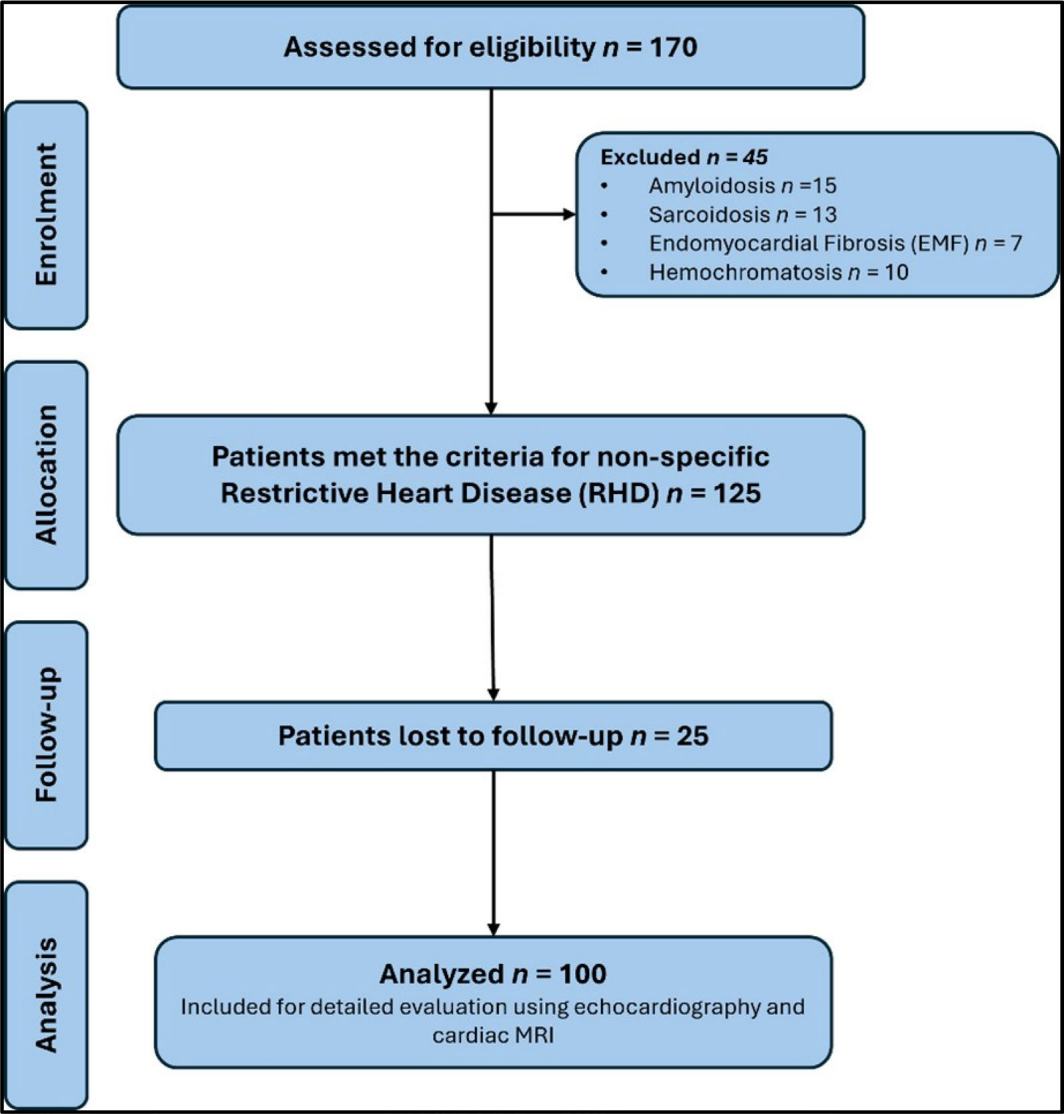
Study population flow chart and analysis framework

This retrospective cohort study was carried out on 100 patients diagnosed with idiopathic restrictive heart disease at Cairo University Hospitals, Egypt. Figure 3

The study cohort (N = 100) demonstrated a mean age of 55.73 ± 11.19 years with a male predominance (58%). BSA was 1.96 ± 0.25 m^2^. CMR findings revealed elevated LAVI and RAVI (LAVI: 62.76 ± 29.80 ml/m^2^, RAVI: 49.41 ± 17.09 ml/m^2^) and preserved mean biventricular EF (LVEF: 55.36 ± 11.79%, RVEF: 52 ± 9.28%). Strain analysis showed moderately impaired GCS (−14.50 ± 4.98%) and GLS (−10.59 ± 4.64%). LGE was present in 40% of patients, and 42% experienced HF hospitalization. The mortality rate was 22% during the study period. Table 1.

**Table 1:** Demographic, echocardiographic, CMR, and outcomes of the studied patients

		N = 100
Age (years)	Mean ± SD	55.73 ± 11.19
	Range	22–85
Gender	Male n (%)	58(58%)
	Female n (%)	42(42%)
BSA(m^2^)	Mean ± SD	1.96 ± 0.25
	Range	0.9–2.8
LA volume (ml)	Mean ± SD	120.17 ± 55.05
	Range	12–288
E/A (ratio)	Mean ± SD	3.2 ± 0.6
	Range	2–5
Deceleration time (ms)	Mean ± SD	147 ± 20
	Range	120–190
IVRT (ms)	Mean ± SD	41 ± 11
	Range	3–65
Average E/e'	Mean ± SD	15 ± 2
	Range	11–18
LAVI (ml/m^2^)	Mean ± SD	62.76 ± 29.80
	Range	13–155
RA volume (ml)	Mean ± SD	94.21 ± 26.42
	Range	41–142
RAVI (ml/m^2^)	Mean ± SD	49.41 ± 17.09
	Range	19–142
LVEDV (ml)	Mean ± SD	133.71 ± 41.09
	Range	51–272
LVEDVI (ml/m^2^)	Mean ± SD	69.71 ± 24.32
	Range	32–183
LVEF (%)	Mean ± SD	55.36 ± 11.79
	Range	19–75
RVEDV (ml)	Mean ± SD	127.94 ± 44.26
	Range	45–280
RVEDVI (ml/m^2^)	Mean ± SD	66.18 ± 23.27
	Range	23–144
RVEF (%)	Mean ± SD	52 ± 9.28
	Range	27–72
GCS (%)	Mean ± SD	− 14.50 ± 4.98
	Range	− 24.1–3.4
GRS (%)	Mean ± SD	29.507 ± 13.46
	Range	3.2–67.2
GLS (%)	Mean ± SD	− 10.59 ± 4.64
	Range	− 22.7–2.8
TR (≥ moderate)	n (%)	11(11%)
LGE (%)	Mean ± SD	4 ± 1
	Range	(1–6)
LGE	n (%)	40 (40%)
HF hospitalization	Yes	42 (42%)
	No	58 (58%)
Mortality	Yes	22 (22%)
	No	78 (78%)

BSA: Body surface area; LA: Left Atrial; HF: Heart Failure; TR: Tricuspid Regurgitation; RA: Right Atrial; RAVI: Right Atrial Volume Index; LAVI: Left Atrial Volume Index; LVEDV: Left Ventricular End-Diastolic Volume;; LGE: Late Gadolinium Enhancement, GCS: Global Circumferential Strain; RVEDV: Right Ventricular End-Diastolic Volume; RVEDVI: Right Ventricular End-Diastolic Volume Index; RSR: Radial Strain Rate; LVEDVI: Left Ventricular End-Diastolic Volume Index; RVEF: Right Ventricular Ejection Fraction; GRS: Global Radial Strain; GLS: Global Longitudinal Strain; LSR: Longitudinal Strain Rate; CSR: Circumferential Strain Rate; LVEF: Left Ventricular Ejection Fraction

Analysis revealed significant associations between mortality and several clinical and imaging parameters. Deceased patients had higher RA volume (105.04 ± 23.75 vs. 91.15 ± 26.46 ml, *p* = 0.028), impaired strain parameters (GCS: −12.29 ± 5.57 vs. −15.15 ± 4.64%, *p* = 0.017; GRS: 23.04 ± 13.50 vs. 31.34 ± 12.95%, *p* = 0.009; GLS: −7.61 ± 2.72 vs. −11.43 ± 4.736%, *p* = 0.004), and greater prevalence of ≥ moderate TR (31.81% vs. 5.12%, *p* < 0.001). Additionally, LGE (81.81% vs. 28.20%, *p* < 0.001) and HF hospitalization (86.36% vs. 29.48%, *p* < 0.001) were significantly more common among deceased patients. Table 2.

**Table 2:** Relation between mortality and all parameters of the studied patients

		Deceased (n = 22)	Alive (n = 78)	*p* value
E/A (ratio)	Mean ± SD	3.1 ± 0.5	3.2 ± 0.6	0.669
	Range	(2.3–4.1)	(2–5)	
Deceleration time (ms)	Mean ± SD	142 ± 20	149 ± 20	0.155
	Range	(120–180)	(120–190)	
IVRT (ms)	Mean ± SD	40 ± 15	41 ± 10	0.646
	Range	(4–65)	(3–60)	
Average E/e'	Mean ± SD	15 ± 2	15 ± 2	0.934
	Range	(11–17)	(11–18)	
LA volume (ml)	Mean ± SD	128.13 ± 51.42	117.92 ± 56.14	0.445
	Range	46–261	12–288	
LAVI (ml/m^2^)	Mean ± SD	66.63 ± 27.03	61.66 ± 30.61	0.492
	Range	30–134	13–155	
RA volume (ml)	Mean ± SD	105.04 ± 23.75	91.15 ± 26.46	0.028*
	Range	43–142	41–142	
RAVI(ml/m^2^)	Mean ± SD	54.59 ± 12.36	47.94 ± 18.05	0.107
	Range	21–73	19–142	
LVEDV (ml)	Mean ± SD	125.90 (± 38.98)	135.91 ± 41.64	0.315
	Range	65–210	51–272	
LVEDVI(ml/m^2^)	Mean ± SD	66.13 ± 23.34	70.71 ± 24.65	0.438
	Range	33–113	32–183	
LVEF (%)	Mean ± SD	53.72 ± 14.29	55.82 ± 11.05	0.465
	Range	19–75	23–75	
RVEDV (ml)	Mean ± SD	117.54 ± 48.26	130.87 ± 42.94	0.214
	Range	45–240	45–280	
RVEDVI (ml/m^2^)	Mean ± SD	60.81 ± 24.83	67.69 ± 22.75	0.222
	Range	30–127	23–144	
RVEF (%)	Mean ± SD	48.72 ± 9.46	52.92 ± 9.08	0.060
	Range	37–72	27–68	
GCS (%)	Mean ± SD	− 12.29 ± 5.57	− 15.15 ± 4.64	0.017*
	Range	− 23.3–3.4	− 24.1–3.4	
GRS (%)	Mean ± SD	23.04 ± 13.50	31.34 ± 12.95	0.009*
	Range	3.2–51.3	3.2–67.2	
GLS (%)	Mean ± SD	− 7.61 ± 2.72	− 11.43 ± 4.736	0.004*
	Range	− 15.6–3.4	− 22.7–2.8	
TR (≥ moderate)		7(31.81%)	4(5.12%)	< 0.001*
LGE	Yes	18(81.81%)	22(28.20%)	< 0.001*
	No	4(18.18%)	56(71.79%)	
HF hospitalization	Yes	19(86.36%)	23(29.48%)	< 0.001*
	No	3(13.63%)	55(70.51%)	

The data yielding a p-value of less than or equal 0.05 is considered statistically significant and is written/marked in bold

BSA: Body Surface Area; LVEDVI: Left Ventricular End-Diastolic Volume Index; LA volume: Left Atrial Volume; LAVI: Left Atrial Volume Index; RA volume: Right Atrial Volume; GLS: Global Longitudinal Strain; RAVI: Right Atrial Volume Index; LVEDV: Left Ventricular End-Diastolic Volume; RVEDV: Right Ventricular End-Diastolic Volume; RVEF: Right Ventricular Ejection Fraction; GCS: Global Circumferential Strain; GRS: Global Radial Strain; LSR: Longitudinal Strain Rate; LVEF: Left Ventricular Ejection Fraction; CSR: Circumferential Strain Rate; RSR: Radial Strain Rate; RVEDVI: Right Ventricular End-Diastolic Volume Index; TR: Tricuspid Regurgitation; LGE: Late Gadolinium Enhancement; HF hospitalization: Hospitalization for Heart Failure; P value < 0.05: Statistically Significant

No significant findings were found regarding LGE % according to mortality or HF hospitalization (Table 3). Univariate regression identified significant predictors of mortality, including RA volume (OR: 1.0217, *p* = 0.032), GCS (OR: 1.1306, *p* = 0.021), GRS (GRS, OR: 0.9451, *p* = 0.013), GLS (OR: 1.2633, *p* < 0.001), ≥ moderate TR (OR: 5.475, *p* = 0.01), LGE (OR: 11.4545, *p* < 0.001), and HF hospitalization (OR: 15.1449, *p* < 0.001). In multivariate regression, GLS (OR: 1.195, *p* = 0.044), LGE (OR: 6.340, *p* = 0.004), and HF hospitalization (OR: 8.961, *p* = 0.001) remained independent predictors of mortality Table 4.

**Table 3:** Relation between HF hospitalization and mortality with LGE (%)

		HF Hospitalization		*p*-value
		Yes (n = 27)	No (n = 13)	
LGE (%)	Mean ± SD	5 ± 1	4 ± 1	0.208
	Range	(2—6)	(1—6)	
		Mortality		
		Deceased (n = 18)	Alive (n = 22)	
	Mean ± SD	5 ± 1	4 ± 1	0.432
	Range	(2—6)	(1—6)	

HF: Hear Failure; LGE: Late Gadolinium Enhancement. *p* value < 0.05: Statistically Significant

**Table 4:** Univariate and multivariate regression for prediction of incidence of mortality

	Univariate regression			Multivariate regression		
	OR	95% CI	*p* value	OR	95% CI	*p* value
RA volume (ml)	1.0217	1.001–1.042	0.032*	1.003	0.980–1.027	0.743
GCS (%)	1.1306	1.018–1.254	0.021*	1.024	0.869–1.207	0.770
GRS (%)	0.9451	0.903–0.988	0.013*	0.984	0.915–1.057	0.661
GLS (%)	1.2633	1.093–1.459	< 0.001*	1.195	1.0041–1.422	0.044*
TR (≥ mod)	5.475	1.485–20.176	0.01*	1.864	0.382–9.080	0.440
LGE	11.4545	3.483–37.666	< 0.001*	6.340	1.770–22.710	0.004*
HF Hospitalization	15.1449	4.081–56.204	< 0.001*	8.961	2.263–35.470	0.001*

The data yielding a p-value of less than or equal 0.05 is considered statistically significant and is written/marked in bold

^*^Significant as *p* value ≤ 0.05, GRS: global radial strain, LGE: late gadolinium enhancement, OR: Odds Ratio, GLS: Global longitudinal strain, TR: Tricuspid regurgitation, GCS: global circumferential strain, CI: Confidence interval, HF: Heart failure

Univariate regression analysis identified significant predictors of HF hospitalization, including RA volume (OR: 1.024, *p* = 0.005), RAVI (OR: 1.031, *p* = 0.026), GLS (OR: 1.195, *p* = 0.008), ≥ moderate TR (OR: 7.636, *p* = 0.012), and LGE (OR: 6.230, *p* < 0.001). In multivariate regression, GLS (OR: 1.152, *p* = 0.013) and LGE (OR: 4.654, *p* = 0.001) remained independent predictors of HF hospitalization. While ≥ moderate TR approached significance (OR: 5.253, *p* = 0.054), its predictive value was not confirmed in multivariate analysis Table 5.

**Table 5:** Univariate and Multivariate regression for prediction of HF hospitalization

	Univariate regression			Multivariate regression		
	OR	95% CI	*p* value	OR	95% CI	*p* value
LA volume (ml)	1.005	0.997–1.012	0.174			
LAVI (ml/m^2^)	1.007	0.994–1.021	0.252			
RA volume (ml)	1.024	1.007–1.041	0.005*	1.013	0.984–1.044	0.373
RAVI (ml/m^2^)	1.031	1.003–1.060	0.026*	0.999	0.956–1.044	0.984
LVEDV (ml)	1.007	0.997–1.017	0.149			
LVEDVI (ml/m^2^)	1.012	0.995–1.030	0.148			
LVEF (%)	0.971	0.938–1.006	0.105			
RVEDV (ml)	0.997	0.988–1.006	0.596			
RVEDVI (ml/m^2^)	0.995	0.977–1.012	0.573			
RVEF (%)	0.969	0.928–1.013	0.171			
GCS (%)	1.083	0.996–1.179	0.061			
GRS (%)	0.975	0.944–1.006	0.112			
GLS (%)	1.195	1.0774–1.326	0.008*	1.152	1.029–1.289	0.013*
TR (≥ moderate)	7.636	1.555–37.500	0.012*	5.253	0.970–28.444	0.054
LGE	6.230	2.577–15.063	< 0.001*	4.654	1.804- 12.008	0.001*

The data yielding a p-value of less than or equal 0.05 is considered statistically significant and is written/marked in bold

^*^Significant as *p* value ≤ 0.05, GCS: global circumferential strain, CI: Confidence interval, GRS: global radial strain, OR: Odds Ratio, GLS: Global longitudinal strain, TR: Tricuspid regurgitation, LGE: late gadolinium enhancement, HF: Heart failure

**Fig. 4:**
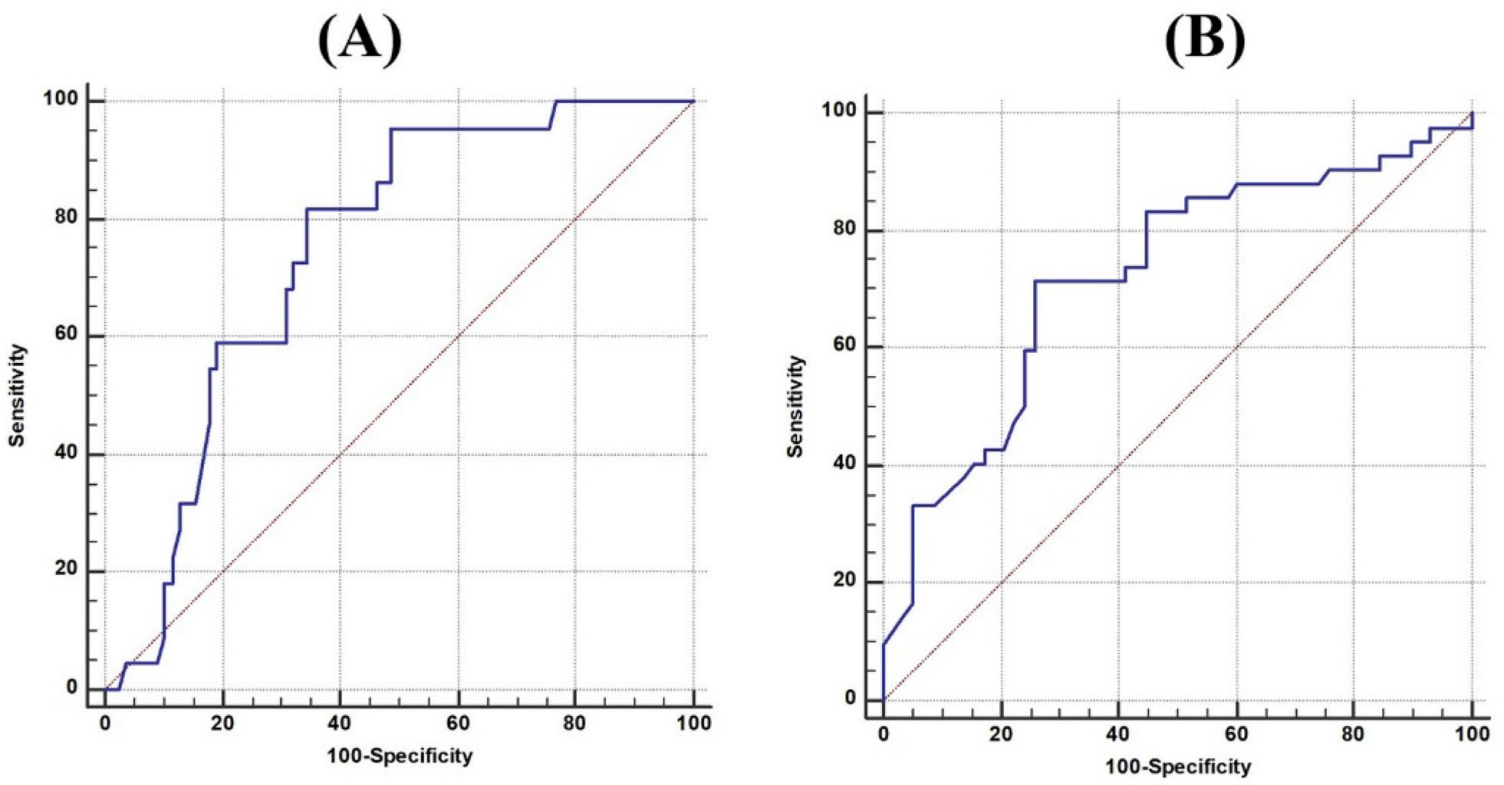
ROC curve of GLS in prediction of incidence of mortality (**A**), and HF hospitalization (**B**)

Table 5 demonstrates the predictive value of GLS for mortality and HF hospitalization. A GLS cutoff of > −9.5 was associated with significant predictive power for both outcomes. For mortality, GLS > −9.5 yielded a specificity of 65.38%, sensitivity of 81.82%, PPV of 40.0%, and NPV of 92.7%, and an AUC of 0.747 (*p* < 0.001). For HF hospitalization, the same cutoff revealed a specificity of 74.14%, sensitivity of 71.43%, PPV of 66.7%, NPV of 78.2%, and an AUC of 0.723 (*p* < 0.001) Table 6, Fig. 4A, and B.

**Table 6:** Role of GLS in prediction of incidence of mortality, and HF hospitalization

Variable	Cut off	Sensitivity	Specificity	PPV (%)	NPV (%)	AUC (95% CI)	*p* value
Prediction of incidence of mortality							
GLS (%)	> -9.5	81.82%	65.38%	40.0	92.7	0.747 (0.701–0.811)	< 0.001*
Prediction of incidence of HF hospitalization							
GLS (%)	> -9.5	71.43%	74.14%	66.7	78.2	0.723 (0.689–0.794)	< 0.001*

NPV: Negative predictive value, AUC: Area under the curve PPV: Positive predictive value, *Significant as *p* value < 0.05

## Discussion

RCM is a form of cardiomyopathy defined by increased myocardial stiffness and compromised ventricular filling, with systolic function remaining relatively preserved until late-stage disease evolution. Diagnostic evaluation typically involves echocardiography, revealing bi-atrial enlargement and preserved systolic function, while CMR offers superior tissue characterization with tools like and LGE [6]. In HFpEF, where LVEF ≥ 50%, strain imaging, particularly GLS, surpasses LVEF in predicting outcomes. Despite CMR’s role as the gold standard for assessing volumes and function, data on CMR-derived strain in HFpEF are limited [7]. This study explores the prognostic value of FT-CMR in idiopathic restrictive heart disease.

In our analysis, the significant association between mortality and higher RA volume suggests that increased atrial size reflects chronic pressure overload and advanced diastolic dysfunction, which are hallmarks of restrictive heart disease. Additionally, moderate or greater TR further exacerbates right-sided volume overload, leading to progressive hemodynamic deterioration.

In consistency with our results, Hong et al. reported in their analysis that moderate or greater TR was an independent predictor of mortality, with HR of 32.55 (CI: 6.69–158.27, *p* < 0.001). These results further support the association between right-sided volume overload and increased mortality risk. [8].

In this study, reduced strain parameters (GCS, GRS, and GLS) were significantly associated with mortality, reflecting impaired myocardial contractility and compliance linked to poor systolic and diastolic function. GLS emerged as an independent predictor of both mortality and HF hospitalization. Reduced GLS, indicative of subendocardial fiber impairment, correlates with myocardial fibrosis and diastolic dysfunction—key features of restrictive heart disease. With a cutoff of > −9.5%, GLS demonstrated moderate diagnostic accuracy for predicting mortality and HF hospitalization.

Similarly, Romano et al. [9] observed that GLS was a significant independent predictor of mortality. Each 1% reduction in GLS was independently associated with a 22.8% greater likelihood of mortality, despite adjustments for both imaging and clinical predictors.

They further demonstrated that GLS remained predictive of mortality even in patients without LGE [9]. Furthermore, Buss et al. [10], over a median follow-up of 5.3 years, found that GLS was a significant independent predictor of adverse outcomes, including heart transplantation, aborted sudden cardiac death, and cardiac death. GLS provided prognostic value beyond that of EF, LGE mass, and NT-proBNP levels. Notably, a GLS greater than -12.5% predicted adverse outcomes even in patients with EF < 35% or the presence of LGE.

Similar to our study, Kalam et al. [11] observed a noteworthy interrelation between all-cause mortality and GLS, with each SD change in GLS predicting a mortality reduction (HR 0.50, 95% CI 0.36 to 0.69; *p* < 0.002). In contrast, LVEF had a weaker association with mortality (HR 0.81, 95% CI 0.72 to 0.92; *p* = 0.572). Furthermore, GLS demonstrated a 1.62 times greater reduction in mortality risk per standard deviation change compared to LVEF. They found that the pooled GLS cut-off for predicting MACE in a general cardiovascular disease population was -14.7%. They included patients with various etiologies and preserved ejection fraction. However, the greater degree of myocardial stiffness and fibrosis in RCM, even with preserved LVEF, may explain why a more severely reduced GLS is associated with adverse outcomes in this subgroup.

Supporting our results, Kammerlander et al. [12] demonstrated that GLS was significantly associated with cardiovascular outcomes, including HF hospitalization and cardiovascular death. A median GLS threshold of > −8.5% was significantly linked to elevated event rates, and multivariable Cox regression analysis identified GLS as an independent prognostic marker of adverse outcomes (HR: 1.06 per 1% strain increase; 95% CI: 1.01–1.11; *P* = 0.03). In consistency with our findings, Gao et al.’s [13] findings demonstrated that LV-GLS progressively declined from controls to RCM patients (all *p* < 0.05). The study also highlighted LGE as an additional predictor of LV deformation. Additionally, they reported the significant association of impaired GLS with adverse outcomes, underscoring its predictive utility.

In contrast to our findings where GLS and LGE emerged as significant predictors, Xiaohui et al.’s multivariate Cox regression analysis demonstrated that age > 65 years (HR = 2.449) and LVEF < 50% (HR = 2.661) were independent predictors of mortality, while parameters like left atrial diameter, atrial fibrillation, and tricuspid regurgitation were not significant. This discrepancy might be attributed to differences in patient populations, as our study utilized CMRI strain analysis, which provides a more nuanced assessment of myocardial deformation and fibrosis. Additionally, the exclusion of ischemic cardiomyopathy and other specific etiologies in our cohort may have contributed to the divergent predictors observed [14].

The predominance of LGE in deceased patients in this study emphasizes the relevance of extensive myocardial fibrosis as a pivotal marker of irreversible structural remodelling and unfavourable cardiac outcomes. Identified as an independent predictor of both HF hospitalization and mortality, LGE reflects the prognostic impact of myocardial fibrosis in restrictive heart disease. It signifies myocardial scarring and fibrotic remodelling, contributing to ventricular stiffness, reduced compliance, and impaired diastolic filling, which exacerbate pressure overload and atrial enlargement, further compromising hemodynamics. From a clinical perspective, LGE is linked to an increased risk of pump dysfunction, arrhythmias, and unfavourable cardiac outcomes.

This is consistent with numerous studies across various cardiomyopathies, where LGE is a robust predictor of adverse outcomes. For instance, Greulich et al. [15] revealed that LGE, present in 25.5% of patients, was the strongest independent predictor of adverse outcomes, including death, aborted sudden cardiac death, and appropriate ICD discharges. The hazard ratios for these endpoints were strikingly high: 31.6 for death or aborted cardiac death and 33.9 for any adverse event. In agreement with our results, Sameer et al. [16] found that LGE patterns (none, subendocardial, and transmural) were strongly associated with amyloid burden, and transmural LGE was an independent predictor of mortality, even after adjusting for clinical and echocardiographic parameters such as NT-proBNP and EF. They also documented a strong correlation between LGE and clinical endpoints such as increased risk of heart failure and arrhythmias.

Furthermore, Baggiano et al. [17] identified LGE as a key CMR indicator of RCM, strongly correlating with myocardial interstitial infiltration. In patients with RCM, the presence of diffuse LGE independently correlated with an elevated risk of late mortality. In concordance with these findings, Wu et al. observed that among patients followed for a median duration of 17 months, 44% (n = 12) of those exhibiting LGE experienced an index composite outcome event, whereas the incidence was markedly lower (8%, n = 3) in those without LGE (*p* < 0.001, Kaplan–Meier survival curves).

Upon adjustment for LV volume index and functional class, the presence of LGE remained a strong predictor, conferring an eight-fold higher likelihood of the primary outcome (HR: 8.2, 95% CI: 2.2–30.9, *p* = 0.002) [18].

In a similar vein, Hombach et al. [19] found that LGE was significantly associated with a worse prognosis in univariate analysis, as adverse events occurred more frequently in patients with LGE than in those without it (HR = 2.26, 95% CI: 1.03–4.99).

A significant distinction was observed in Kaplan–Meier survival curves when comparing patients with LGE to those without, highlighting differential prognostic implications [19]. Supporting these observations, Assomull et al. [20] revealed that mid-wall fibrosis in cardiomyopathy conferred an elevated risk of all-cause mortality and an increased likelihood of cardiovascular hospitalization. LGE continued to demonstrate clinical relevance within the multivariable analysis, which included RVEF, LVEF, LVEDV, and LVESV.

Supporting our results, Ismail et al. [21] found that LGE was observed in 70% of cases, showing varied patterns, including mid-wall patchy, subendocardial, and transmural enhancement, indicative of secondary RCM. The study concluded that LGE-CMR is a reliable tool to distinguish between primary and secondary RCM and guide prognosis and management.

This study is subject to several limitations. While the sample size was adequate for statistical analysis, its relatively small number of participants may reduce the reliability of subgroup analyses and constrain the generalizability of certain findings. Additionally, the study did not differentiate between various etiologies of restrictive heart disease, as no genetic or specific etiological investigations were performed. External validation is warranted, however, given the rarity of idiopathic RCM, such validation would ideally be addressed through multicenter collaboration or registry-based studies.

## Conclusions

FT-CMR is a valuable tool for prognostication in idiopathic restrictive heart disease, with myocardial strain parameters and the presence of myocardial fibrosis (LGE) identified as independent predictors of adverse outcomes, including mortality and heart failure hospitalization.

## Data Availability

No datasets were generated or analysed during the current study.

## Abbreviations

RCM: Restrictive cardiomyopathy
CMR: Cardiac magnetic resonance
LGE: Late gadolinium enhancement
HF: Heart failure
LV: Left ventricle / left ventricular
TDI: Tissue doppler imaging
GLS: Global longitudinal strain
EF: Ejection fraction
HFpEF: Heart failure with preserved ejection fraction
LVEF: Left ventricular ejection fraction
GRS: Global radial strain
RV: Right ventricle / right ventricular
GCS: Global circumferential strain
bSSFP: Balanced steady-state free precession
eGFR: Estimated glomerular filtration rate
LVEDVI: Left ventricular end-diastolic volume index
TR: Tricuspid regurgitation
BSA: Body surface area
LA: Left atrium / left atrial
LAVI: Left atrial volume index
RA: Right atrium / right atrial
E/A Ratio: Early-to-late diastolic flow velocities ratio
RAVI: Right atrial volume index
LSR: Longitudinal strain rate
RVEDV: Right ventricular end-diastolic volume
OR: Odds ratio
LVEDV: Left ventricular end-diastolic volume
CSR: Circumferential strain rate
RSR: Radial strain rate
CI: Confidence interval
RVEDVI: Right ventricular end-diastolic volume index
PPV: Positive predictive value
NT-proBNP: N-terminal pro B-type natriuretic peptide
NPV: Negative predictive value
LVESV: Left ventricular end-systolic volume
AUC: Area under the curve
ROC: Receiver operating characteristic
HR: Hazard ratio
ICD: Implantable cardioverter-defibrillator
